# The Bacillus of Scarlatina

**Published:** 1888-02

**Authors:** 


### THE BACILLUS OF SCARLATINA.

The report of the committee appointed by the Edinburgh Medico-Chirurgical Society to investigate the organisms of scarlatina, although not a final one, nevertheless contains some interesting facts. The most important of these is the doubt expressed by the committee as to whether calves are at all susceptible to the scarlatinal poison. In conjunction with Professor MacFadyean they injected scarlatinal blood into two calves, and fed a third with desquamated scales. All three experiments gave negative results. This observation is an important one, and it is to be hoped that the experiments on this subject will be continued. For it will be remembered that the keystone of Dr. Klein's research is the assumption that cattle can take scarlatina, while both Dr. Klein and Mr. Edington lay some stress on the results of the inoculations of their organisms on calves. Mr. Edington inoculated two calves, one of which died within twenty-four hours, and the other, which was only one day old, recovered after a short ilness, in which the prominent symptons were high temperature, inflamed throat, and redness of the skin, followed by desquamation. If the committee definitely show that cattle of every age are incapable of being affected by the scarlatinal poison, the assumption on which Drs. Power and Klein base their views falls to the ground, while the result of Mr. Edington's experiment tells rather against than in favor of the idea that his bacillus is the cause of human scarlatina.

A second point of importance is the statement that the micro-oganism described by Mr. Edington under under the name of streptococcus rubiginosus is morphologically identical with the streptococcus scarlatinæ of Klein. According to Mr. Edington, this organism is not present in the blood in the early stage of the disease, and its inoculation into rabbits, guinea-pigs, and pigs gave negative results. Whether, though morphologically identical with Dr. Klein's organisms, it is really the same, has not yet been determined. In connection with this microbe, the fact stated by Mr. Edington in his letter to the committee is interesting, namely, that his brother drank a cultivation of this organism thirty-six hours old, and made directly from scarlatinal blood taken on the fourth day of the disease without showing any bad result, although the streptococcus rubiginosus was found in his blood three days later, and although he had never had scarlet fever.

It is also interesting to note that the results of the microscopial examinations and cultivations made from the blood were by no means constant. In ten cases of scarlet fever examined microscopically on or before the third day, bacilli and micrococci together were found in three cases, micrococci alone in four, and no micro-organisms in the remaining three. The results obtained by cultivation were very similar; in four out of nine cases bacilli and micrococci appeared in the culture material, in two micrococci alone, and in three the result was negative.

The committtee also express their belief that Mr. Edington's bacillus scarlatinæ is not identical with the bacillus recently found by Dr. Smith in the skin, and supposed by him to be the same, nor with the bacillus butyris, nor the bacillus subtilis. It is to be hoped that the commitee will continue their investigations on this interesting and important subject.—*British Medical Journal.*

